# Supplementary material for: Mitochondrial, lysosomal and DNA damages induced by acrylamide attenuate by ellagic acid in human lymphocyte
Source: PLoS One. 2021 Feb 26;16(2):e0247776. doi: 10.1371/journal.pone.0247776 (PMC7909646; doi:10.1371/journal.pone.0247776)
Supplement: Attachment — Submitted filename: Resoponse to reviewers.doc [file pone.0247776.s010.doc]

Prof. Soumen Bera, PhD

Academic Editor

PLOS ONE

Dear Prof. Bera,

Thank you very much for your letter of 8-Jan-2021, regarding the reviewers' comments on our submitted manuscript PONE-D-20-36110 entitled " Mitochondrial, Lysosomal and DNA Damages Induced by Acrylamide Attenuate by Ellagic Acid in Human Lymphocyte ". We carefully read the reviewers' comments and included their requests in our revised article and improved it according to their wish. I hope it is now satisfactory. In the following, please see our answers to the reviewers' comments point by point.

In the end, I would like to thank you and the respected reviewers for dedicating precious time and offering scientific comments.

With kind regards

Ahmad Salimi

**Comments to the Author**

1. Is the manuscript technically sound, and do the data support the conclusions?

The manuscript must describe a technically sound piece of scientific research with data that supports the conclusions. Experiments must have been conducted rigorously, with appropriate controls, replication, and sample sizes. The conclusions must be drawn appropriately based on the data presented.

Reviewer #1: Yes

Reviewer #2: Yes

2. Has the statistical analysis been performed appropriately and rigorously?

Reviewer #1: Yes

Reviewer #2: Yes

3. Have the authors made all data underlying the findings in their manuscript fully available?

The [PLOS Data policy](http://www.plosone.org/static/policies.action#sharing) requires authors to make all data underlying the findings described in their manuscript fully available without restriction, with rare exception (please refer to the Data Availability Statement in the manuscript PDF file). The data should be provided as part of the manuscript or its supporting information, or deposited to a public repository. For example, in addition to summary statistics, the data points behind means, medians and variance measures should be available. If there are restrictions on publicly sharing data—e.g. participant privacy or use of data from a third party—those must be specified.

Reviewer #1: Yes

Reviewer #2: Yes

4. Is the manuscript presented in an intelligible fashion and written in standard English?

PLOS ONE does not copyedit accepted manuscripts, so the language in submitted articles must be clear, correct, and unambiguous. Any typographical or grammatical errors should be corrected at revision, so please note any specific errors here.

Reviewer #1: No

Reviewer #2: Yes

**Our response:** As requested by respected reviewer we checked the whole manuscript for typographical or grammatical errors and corrected them. Please refer to revised version and see yellow bands in the revised manuscript.

5. Review Comments to the Author

Please use the space provided to explain your answers to the questions above. You may also include additional comments for the author, including concerns about dual publication, research ethics, or publication ethics. (Please upload your review as an attachment if it exceeds 20,000 characters)

Reviewer #1: The manuscript by Baghal et al. describes the consequences of the exposure to human lymphocytes to acrylamide and the protection offered to those cells by the simultaneous treatment with ellagic acid. Acrylamide toxicity has been extensively studied, but both the use of primary human lymphocytes and description of the protection offered by ellagic acid are novel results. Several different endpoints where considered and the data supports the conclusions that ellagic acid could prevent mitochondrial damage and oxidative stress due to acrylamide exposure. The data is presented concisely, and the experiments were performed carefully. However, the manuscript would benefit from editing to improve grammar and wording as well as correct typographical errors. Specific issues are presented below:

1. Several qualities of acrylamide are provided by the manufacturer and it should be specified which was obtained for the described experiments.

**Our response:** As requested by respected reviewer we added the CAS number acrylamide in the materials and methods in the chemical and kits section. Please refer to revised version and see yellow bands in the revised manuscript.

2. Some additional information regarding participants in the study, such as gender and how were the healthy individuals recruited, would be useful.

**Our response:** As requested by respected reviewer we added additional information regarding participants in the study. Please refer to revised version and see yellow bands in the revised manuscript.

3. The specific software used for the reported analyses should be specified in the Methods section.

**Our response:** As requested by respected reviewer we added the necessary information for statistical software and other information about statistical analysis methods in the text. Please refer to revised version and see yellow bands in the revised manuscript.

4. The oxidative lesion 8-OHdG often occurs during handling and storage. Where there any specific precautions taken to limit this from happening? Also, the production of 8-OHdG was not formally determined, just the steady state levels. It is possible that it was the removal of the lesion that was affected by the treatments.

**Our response:** Thank you very much for your good question, in the current study we measured 8-OHdG as DNA Damage indicator exactly after 4 hours of exposure and compared with untreated control for remove of other interventions. Also, we used the standard protocol for detection of 8-OHdG according previous studies.

5. The Discussion could be shortened to provide less general background and instead focus on the significance and implications of the data obtained.

**Our response:** Thank you very much for your good comment, as requested by respected reviewer we shorted the Discussion and focus on the significance and implications of the data obtained as much as possible and added the limitation of the study. Please refer to revised version and see yellow bands in the revised manuscript.

6. In the References, reference 1 lacks details and the formatting of many references requires correction.

**Our response:** Thank you very much for your good comment, as requested by respected reviewer we corrected the reference 1. Please refer to revised version and see yellow bands in the revised manuscript.

Reviewer #2: Comment
The work presented provides evidence that ellagic protects lymphocytes from the toxic effects of ellagic acid. The authors show that several end points for mitochondrial function and oxidative stress induced by acrylamide are inhibited by ellagic acid.

1. The author need to provide more physiological evidence for there findings in a cell or animal model to support the in vitro lymphocyte data presented.

**Our response:** Thank you very much for your comment as you know we used the isolated human lymphocytes in this study as wild type cells and useful model for assessment and evaluation of toxicity induced by drug and chemicals, in here we showed that ellagic acid with antioxidant potential reduced the toxic effect of ellagic acid. We used BHT as a known antioxidant for confirmation (data not shown in the graphs). Due to the fact that this study was performed on primary human lymphocytes, it is not suitable for animal models. As main limitation in the current study we suggested in the last paragraph in the revised manuscript. Please refer to revised version and see yellow bands in the revised manuscript.

2. Can the authors provided evidence for the mechanism of ellagic acid and how it blocks the toxicity of acrylamide?

**Our response:** In the current study we used the butylated hydroxytoluene (BTH) as a known antioxidant for confirmation of this fat that ellagic acid through antioxidant potential reduce the acrylamide-induced toxicity in human lymphocytes. As requested by respected reviewer we added the details in the revised version. Please refer to revised version and see yellow bands in the revised manuscript.

3. Is the protection by ellagic acid related to its antioxidant properties or some other mechanism?

**Our response:** Yes. Please see above our responses (In the current study we used the butylated hydroxytoluene (BTH) as a known antioxidant for confirmation of this fat that ellagic acid through antioxidant potential reduce the acrylamide-induced toxicity in human lymphocytes).

6. PLOS authors have the option to publish the peer review history of their article ([what does this mean?](https://journals.plos.org/plosone/s/editorial-and-peer-review-process#loc-peer-review-history)). If published, this will include your full peer review and any attached files.


If you choose “no”, your identity will remain anonymous but your review may still be made public.


**Do you want your identity to be public for this peer review?** For information about this choice, including consent withdrawal, please see our [Privacy Policy](https://www.plos.org/privacy-policy).

Reviewer #1: No

Reviewer #2: No
